# Ticagrelor vs Prasugrel for Acute Coronary Syndrome in Routine Care

**DOI:** 10.1001/jamanetworkopen.2024.48389

**Published:** 2024-12-02

**Authors:** Nils Krüger, Johannes Krefting, Thorsten Kessler, Raphael Schmieder, Fabian Starnecker, Alexander Dutsch, Christian Graesser, Ulrike Meyer-Lindemann, Theresa Storz, Irina Pugach, Christian Frieß, Zhifen Chen, Dario Bongiovanni, Iulian Manea, Tobias Dreischulte, Frank Offenborn, Peter Krase, Hendrik B. Sager, Jens Wiebe, Sebastian Kufner, Erion Xhepa, Michael Joner, Teresa Trenkwalder, Ulrich Gueldener, Adnan Kastrati, Salvatore Cassese, Heribert Schunkert, Moritz von Scheidt

**Affiliations:** 1 Department of Cardiology, German Heart Center Munich, Technical University of Munich, Munich, Germany; 2 Deutsches Zentrum für Herz-Kreislauf-Forschung, Partner Site Munich Heart Alliance, Munich, Germany; 3 Department of Internal Medicine I, Cardiology, University Hospital Augsburg, University of Augsburg, Augsburg, Germany; 4 School of Electronic Engineering and Computer Science, Queen Mary University of London, London, United Kingdom; 5 Institute of General Practice and Family Medicine, LMU University Hospital, LMU Munich, Munich, Germany; 6 Allgemeine Ortskrankenkasse Bayern, Munich, Germany

## Abstract

**Importance:**

In patients with acute coronary syndrome (ACS) undergoing invasive treatment, ticagrelor and prasugrel are guideline-recommended P2Y12 receptor inhibitors. The ISAR-REACT5 randomized clinical trial demonstrated superiority for prasugrel, although concerns were raised about the generalizability of some underpowered subgroup analyses.

**Objectives:**

To emulate a randomized clinical trial evaluating the safety and effectiveness of ticagrelor vs prasugrel under the conditions of routine care in individuals with ACS planned to undergo an invasive treatment strategy.

**Design, Setting, and Participants:**

This new-user cohort study included secondary data from a German statutory health insurance claims database between January 2012 and December 2021, using 1:1 propensity score nearest-neighbor matching to emulate ISAR-REACT5. Individuals with ACS receiving either ticagrelor or prasugrel treatment after hospital discharge were followed up for 1 year. Eligibility criteria closely emulated those of ISAR-REACT5 and included age of 18 years or older and cardiovascular risk factors. Data were analyzed from May 2023 to May 2024.

**Exposure:**

Outpatient prescription of ticagrelor or prasugrel.

**Main Outcomes and Measures:**

The primary end point was the composite of all-cause mortality, myocardial infarction (MI), or stroke within 1 year of outpatient treatment initiation. Secondary end points included individual components of the primary end point and stent thrombosis. The safety end point was major bleeding. A Cox proportional hazards regression model was fitted to the overall cohort.

**Results:**

Of 17 642 propensity score–matched individuals (mean [SD] age, 63.1 [10.9] years; 73.9% male), 8821 received ticagrelor and 8821 received prasugrel. Agreement was met in 11 of 12 predefined agreement metrics when comparing the results with ISAR-REACT5. The primary composite end point of all-cause mortality, MI, or stroke occurred in 815 individuals (9.2%) receiving ticagrelor and 663 (7.5%) receiving prasugrel (hazard ratio [HR], 1.24; 95% CI, 1.12-1.37). Myocardial infarction (HR, 1.20; 95% CI, 1.06-1.36) and stroke (HR, 1.33; 95% CI, 1.02-1.74) each occurred significantly more often in the ticagrelor group. Analysis of all-cause mortality (HR, 1.27; 95% CI, 0.99-1.64), stent thrombosis (HR, 1.11; 95% CI, 0.89-1.30), and major bleeding (HR, 1.12; 95% CI, 0.96-1.32) revealed no significant differences between treatment groups. Subgroup analysis showed that prasugrel was associated with the primary composite end point in fewer individuals with ST-segment elevation MI (338 of 4941 [6.8%] vs 451 of 4852 [9.3%]).

**Conclusions and Relevance:**

This cohort study found that prasugrel was associated with lower rates of all-cause mortality, MI, or stroke compared with ticagrelor in individuals with ACS undergoing an invasive treatment strategy in routine care, particularly in individuals with ST-segment elevation MI. The findings suggest that carefully designed database studies can complement and extend findings from randomized clinical trials, informing guidelines and clinical decision-making.

## Key Points

**Question:**

In routine clinical care, when compared with ticagrelor, is prasugrel associated with improved cardiovascular outcomes among individuals with acute coronary syndrome?

**Findings:**

In this cohort study of 17 642 propensity score–matched individuals, compared with ticagrelor, prasugrel was associated with a significantly lower incidence of the primary composite end point of all-cause mortality, myocardial infarction, or stroke.

**Meaning:**

In routine care, prasugrel was associated with improved outcomes, supporting its preferred use in the treatment of acute coronary syndrome in individuals undergoing an invasive treatment strategy.

## Teaser

This cohort study compares the cardiovascular and major bleeding outcomes of prasugrel vs ticagrelor among new users undergoing invasive treatment for acute coronary syndrome.

## Introduction

The optimal treatment choice between the P2Y12 receptor inhibitors ticagrelor and prasugrel for acute coronary syndrome (ACS) remains debated in the context of dual-antiplatelet therapy, as randomized clinical trials (RCTs) comparing the 2 agents are limited and were inconclusive.^1,2,3^ The Intracoronary Stenting and Antithrombotic Regimen: Rapid Early Action for Coronary Treatment–5 (ISAR-REACT5 [IR5]) RCT^1^ compared ticagrelor and prasugrel for treatment of ACS in patients planned for an invasive treatment strategy. Prasugrel demonstrated superior efficacy over ticagrelor in the reduction of the primary composite end point of all-cause mortality, nonfatal myocardial infraction (MI), or nonfatal stroke.^1^ For the safety end point, defined as major bleeding, the trial observed no statistically significant difference between the 2 agents.

The Comparison of Prasugrel and Ticagrelor in the Treatment of Acute Myocardial Infarction (PRAGUE-18) RCT compared ticagrelor with prasugrel in patients with acute MI.^2,3^ Its primary composite end point (all-cause death, MI, stroke, major bleeding requiring transfusion, or prolongation of hospitalization) did not differ significantly between the agents at the 7-day or 1-year follow-up. However, interpretation was limited due to underpowering, with approximately 40% of the study population switching to clopidogrel after hospital discharge likely due to patients having to cover the costs of the more expensive study medication.

Subsequent to the trials, in 2023, the European Society of Cardiology implemented a modest guideline recommendation favoring prasugrel over ticagrelor for patients with ACS who proceed to percutaneous coronary intervention (PCI) (class IIa, level B).^4^ In contrast, the American College of Cardiology and American Heart Association guideline recommends both drugs equally in combination with aspirin.^5^

Regulatory agencies in Europe and the US increasingly recognize and reevaluate the value of evidence derived from observational data to guide regulatory decision-making^6,7,8^ by overseeing and promoting comparative analyses.^9,10^ Given the ongoing debate and limited evidence for the optimal treatment choice between ticagrelor and prasugrel in the treatment of ACS,^11^ we aimed to estimate the causal effects of the 2 drugs using observational data from insurance claims. By emulating IR5, we aimed to evaluate whether the evidence generated in the present study would support the same regulatory and clinical conclusions as the trial.

## Methods

### Study Design

In this study, a new-user cohort design was used with the aim of emulating IR5^1^ as closely as possible in claims data by creating observational analogues for the population, intervention, comparator, outcome, and timing (PICOT) criteria that define the trial’s research question. This included aligning time 0 with the target trial. In IR5, cohort entry was the day that patients were randomized during hospitalization. However, as claims data do not include information on inpatient medication, cohort entry was therefore defined as the time of the first observed outpatient initiation of ticagrelor or prasugrel after ACS. To account for lack of randomization, 1:1 propensity score nearest-neighbor matching was performed on preexposure characteristics measured in the year prior to the ACS event.^12,13^ These characteristics were compiled from IR5 baseline information,^1^ potential confounders from the literature,^1,14,15^ and the investigators’ clinical expertise. They included baseline demographics, cardiovascular risk factors, ACS type, medical procedure type, and time of ACS (eTable 3 in Supplement 1). A study protocol, including a detailed analysis plan for emulation of the trial eligibility criteria, was developed a priori before analyses were conducted and was registered at the World Health Organization’s linked German Registry of Clinical Studies to allow a structured, transparent, and reproducible implementation process. Approval for the current study was granted by the ethics committee of the Technical University Munich. Informed consent was waived because data were deidentified. We followed the Strengthening the Reporting of Observational Studies in Epidemiology (STROBE) reporting guideline.

### Population

Eligibility criteria of IR5^1^ were emulated closely (eTable 2 in Supplement 1). Inclusion criteria were age of 18 years or older and an *International Statistical Classification of Diseases, Tenth Revision, German Modification (ICD-10-GM)* code for ACS (ST-segment elevation MI [STEMI], non-STEMI [NSTEMI], or unstable angina [UA]). These codes were internally validated by requiring an invasive assessment with diagnostic cardiac catheterization, PCI, or coronary artery bypass graft surgery (CABG) within 3 days of ACS diagnosis. Exclusion criteria reflecting the target trial were applied thereafter (eTable 2 in Supplement 1). Individuals who were dispensed ticagrelor or prasugrel within 180 days before the ACS event were excluded from the study. Furthermore, continuous enrollment in the database for at least 12 months prior to study entry was required to assess baseline covariates.

### Intervention, Comparator, Outcome, and Timing

Individuals were assigned to their respective treatment group according to their first observed out-of-hospital prescription fill for ticagrelor or prasugrel no later than 14 days after an ACS event. Emulating IR5, the primary end point was the composite of all-cause mortality, MI, or stroke. Secondary end points were individual components of the primary end point as well as stent thrombosis. The safety end point was major bleeding. All outcome variables are provided in eTable 1 in Supplement 1. Individuals were followed up from the date of the first observed outpatient prescription of ticagrelor or prasugrel after ACS diagnosis until 1 year.

In alignment with the intention-to-treat analysis used in IR5, all individuals were analyzed according to their treatment assignment regardless of adherence to the study drugs. To assess in-hospital mortality, which was not included in the primary analysis to avoid immortal time, we evaluated mortality in the entire cohort with ACS within the mean duration between ACS diagnosis and the initial outpatient prescription of the P2Y12 receptor inhibitor.

### Data Source and Implementation Process

German claims from the statutory health insurance database Allgemeine Ortskrankenkasse Bayern between January 2012 and December 2021, as part of the Observational Bavarian Health Insurance Registry, were used.^16^ The database contains deidentified demographics and information for covered health care encounters including inpatient and outpatient diagnoses, medical procedures, community-dispensed prescriptions, and vital status information (eTable 1 in Supplement 1).

### Subgroup Analyses

To address subgroups for whom results remained inconclusive in IR5, we performed a detailed analysis on the distinct ACS conditions: STEMI, NSTEMI, and UA. The aim was to identify differential responses to treatment and inform therapeutic strategies accordingly.

### Predefined Binary Agreement Metrics Between Database Study and RCT Results

To evaluate whether the database study would support the same regulatory and clinical conclusions as IR5, 2 predefined binary metrics were used to compare the results. First was regulatory agreement, defined by both the hazard ratio (HR) and 95% CI being on the same side of 1; second was estimate agreement, defined by the HR for the database study falling within the 95% CI of the RCT, as there may have been cases in which the current study found a statistically significant association due to substantially higher power but the RCT failed to find statistical significance.^17^

### On-Treatment Analysis

To mimic high adherence patterns observed in RCTs, an on-treatment design was used. This analysis defined the time from the date of the first observed outpatient initiation of the study drugs to the time of discontinuation, treatment switch, or an end point. Adherence to study medication was assessed indirectly based on pharmacy prescription fills.

### Sensitivity Analysis

To evaluate the robustness of the analysis for the primary end point and address potential residual confounding, preexposure characteristics were extended for propensity score matching. Along with demographics, lifestyle factors, and comorbidities, we also considered disease-specific variables, including the use of cardiovascular and other medications, cardiovascular procedures, and health care utilization indicators. These indicators served as proxies for the overall disease state, care intensity, and surveillance (eTable 4 in Supplement 1).

To assess potential net bias from residual confounding, measurement issues, or follow-up criteria, influenza was used as a negative control outcome. Observed prescription fills for aspirin within 14 days of ACS diagnosis were computed for both groups.

### Statistical Analysis

Propensity score matching was conducted to balance confounders between the treatment groups. We used a 1:1 nearest-neighbor method with a 1% caliper based on the preexposure characteristics (eTables 3 and 4 in Supplement 1).

Kaplan-Meier survival curves were constructed for the treatment groups using the Kaplan-MeierFitter class from the lifelines package in Python, version 3.11.6 (Python Software Foundation).^18^ Respective survival functions were plotted. For nonfatal outcomes, the Aalen-Johansen method, as implemented in the lifelines package, together with the packages survival and cmprsk in RStudio, version 4.1.2 (RStudio, PBC), were used to account for competing risks, as in IR5.^1^

A Cox proportional hazards regression model was fitted to the overall cohort that included follow-up times, censoring indicators, and treatment group. Treatment was included as the only variable of interest. Results were reported as HRs and 95% CIs. The proportional hazards assumption was tested by examining log-log survival plots. Additionally, to statistically assess the validity of the proportional hazards assumption, Schoenfeld residuals were calculated and tested for any correlation with time using the cox.zph function from the survival package in R, version 4.1.2. All analyses were conducted from May 2023 to May 2024 using Python, version 3.11.6^19^ and RStudio, version 4.1.2.^20^

## Results

**Figure 1.**
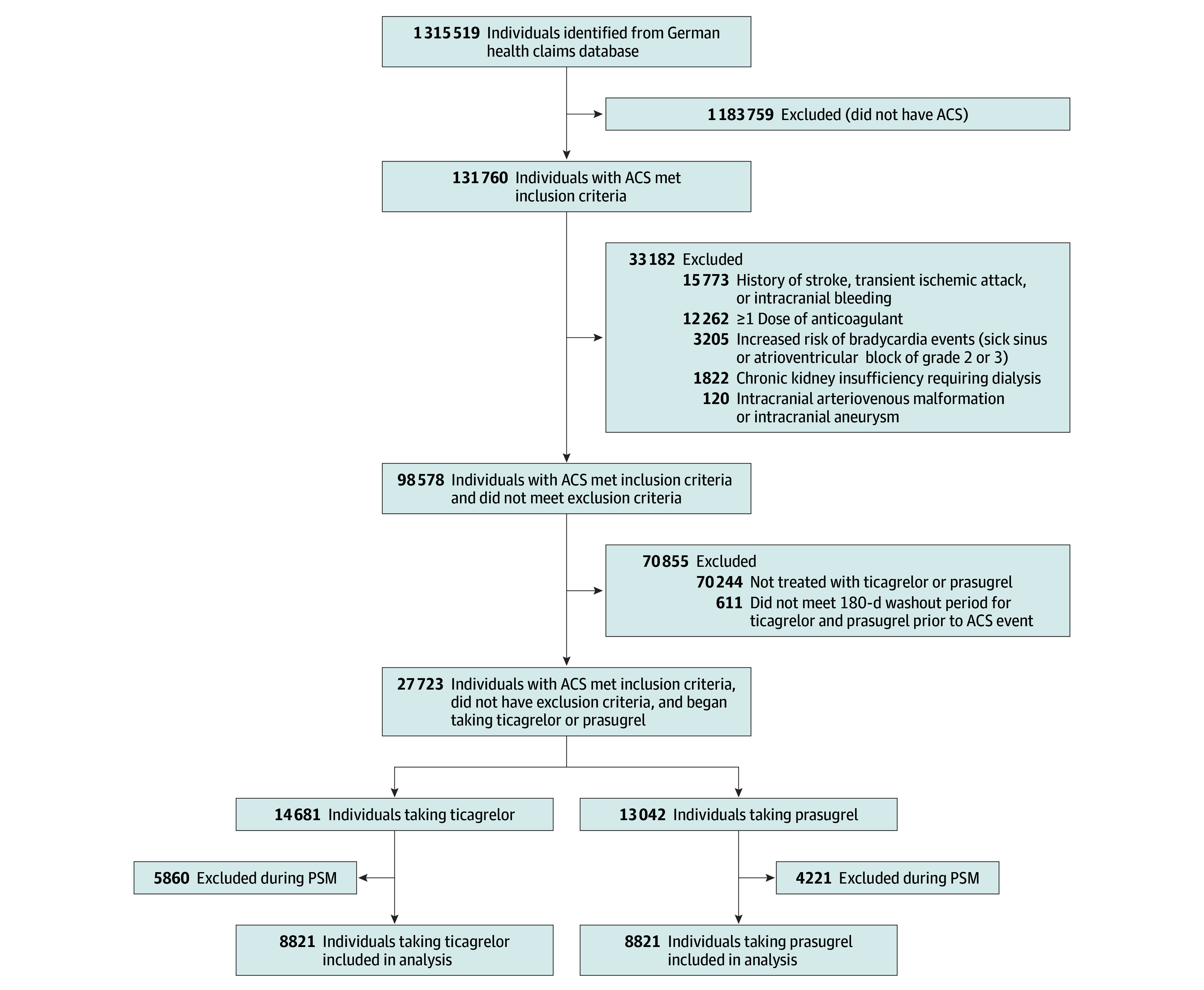
Study Overview of Screening, Eligibility Assessment, and Propensity Score Matching (PSM) Specific *International Statistical Classification of Diseases, Tenth Revision, German Modification (ICD-10-GM)* codes are provided for exclusion criteria. ACS indicates acute coronary syndrome.

Of 131 760 individuals in the database with ACS who underwent an invasive treatment strategy, 27 723 (21.0%) were treated with ticagrelor or prasugrel and met the emulated IR5 eligibility criteria. After propensity score matching, 17 642 matched individuals (8821 in each group) remained (Figure 1, Table 1). Mean (SD) age was 63.1 (10.9) years; 73.9% were men, and 26.1% were women. ACS types included STEMI (9793 individuals [55.5%]), NSTEMI (6558 [37.2%]), and UA (1291 [7.3%]). Individuals were almost exclusively treated with PCI (98.5%); 0.2% received CABG, and 1.3% received conservative treatment. For the ticagrelor group, the median follow-up was 12.0 months (IQR, 12.0-12.0 months; mean [SD], 10.7 [3.2] months); for the prasugrel group, the median follow-up was 12.0 months (IQR, 12.0-12.0 months; mean [SD], 10.8 [3.0] months).

**Table 1.** Baseline Characteristics Before and After Propensity Score Matching

Preexposure characteristic^a^	Before propensity score matching			After propensity score matching		
	Ticagrelor (n = 14 681)^b^	Prasugrel (n = 13 042)^b^	SMD	Ticagrelor (n = 8821)^b^	Prasugrel (n = 8821)^b^	SMD
Age, mean (SD), y	67.5 (12.0)	60.2 (10.5)	0.648	63.1 (11.7)	63.0 (10.0)	0.007
Sex						
Female	4690 (31.9)	2966 (22.7)	−0.208	2292 (26.0)	2316 (26.3)	−0.006
Male	9991 (68.1)	10 076 (77.3)	0.208	6529 (74.0)	6505 (73.7)	0.006
Cardiovascular risk factors						
Diabetes	5412 (36.9)	3993 (30.6)	0.132	2941 (33.3)	2926 (33.2)	0.004
Use of insulin	1250 (8.5)	817 (6.3)	0.086	626 (7.1)	624 (7.1)	0.001
Current smoker	2919 (19.9)	3748 (28.7)	0.208	2213 (25.1)	2196 (24.9)	0.004
Hypertension	12 371 (84.3)	9834 (75.4)	0.222	7036 (79.8)	7087 (80.3)	−0.014
Hyperlipidemia	11 609 (79.1)	9648 (74.0)	0.121	6719 (76.2)	6763 (76.7)	−0.012
Medical history						
Myocardial infarction	2359 (16.1)	1777 (13.6)	0.069	1294 (14.7)	1286 (14.6)	0.003
PCI	586 (4.0)	319 (2.5)	0.088	250 (2.8)	263 (3.0)	0.009
CABG	64 (0.4)	15 (0.1)	0.061	16 (0.2)	14 (0.2)	0.006
Comorbidities						
Chronic kidney disease	2765 (18.8)	1362 (10.4)	0.239	1128 (12.8)	1139 (12.9)	−0.004
Obesity	3659 (24.9)	3062 (23.5)	0.034	2169 (24.6)	2111 (23.9)	0.015
ACS diagnosis at admission						
Unstable angina	1317 (9.00)	706 (5.4)	0.138	655 (7.4)	636 (7.2)	0.007
NSTEMI	7261 (49.5)	3656 (28.0)	0.451	3314 (37.6)	3244 (36.8)	0.003
STEMI	6103 (41.6)	8680 (66.6)	0.518	4852 (55.0)	4941 (56.0)	−0.007
Treatment strategy						
PCI	14 118 (96.2)	12 897 (98.9)	0.176	8694 (98.6)	8684 (98.4)	0.009
CABG	122 (0.8)	15 (0.1)	0.104	17 (0.2)	15 (0.2)	0.005
Conservative therapy	441 (3.0)	130 (1.0)	0.144	110 (1.2)	122 (1.4)	−0.012

Abbreviations: ACS, acute coronary syndrome; CABG, coronary artery bypass grafting; NSTEMI, non–ST-segment elevation myocardial infarction; PCI, percutaneous coronary intervention; SMD, standardized mean difference; STEMI, ST-segment elevation myocardial infarction.

^a^ Preexposure characteristics were measured in the 1 year prior to the ACS event.

^b^ Data are presented as number (percentage) of participants unless otherwise indicated.

Of the 23 inclusion criteria from IR5, the present study was able to directly emulate 13 (56.5%) and indirectly emulate 7 (30.4%); it left 3 (13.0%) unmet. Similarly, of the 19 exclusion criteria, 6 (31.6%) were met directly, 1 (5.3%) was met indirectly, and 12 (63.2%) remained unmet (eTable 2 in Supplement 1). Differences in the measurement of some key study parameters were found when close observational analogues to trial design elements could not be identified due to differences in the type of data collected.

**Figure 2.**
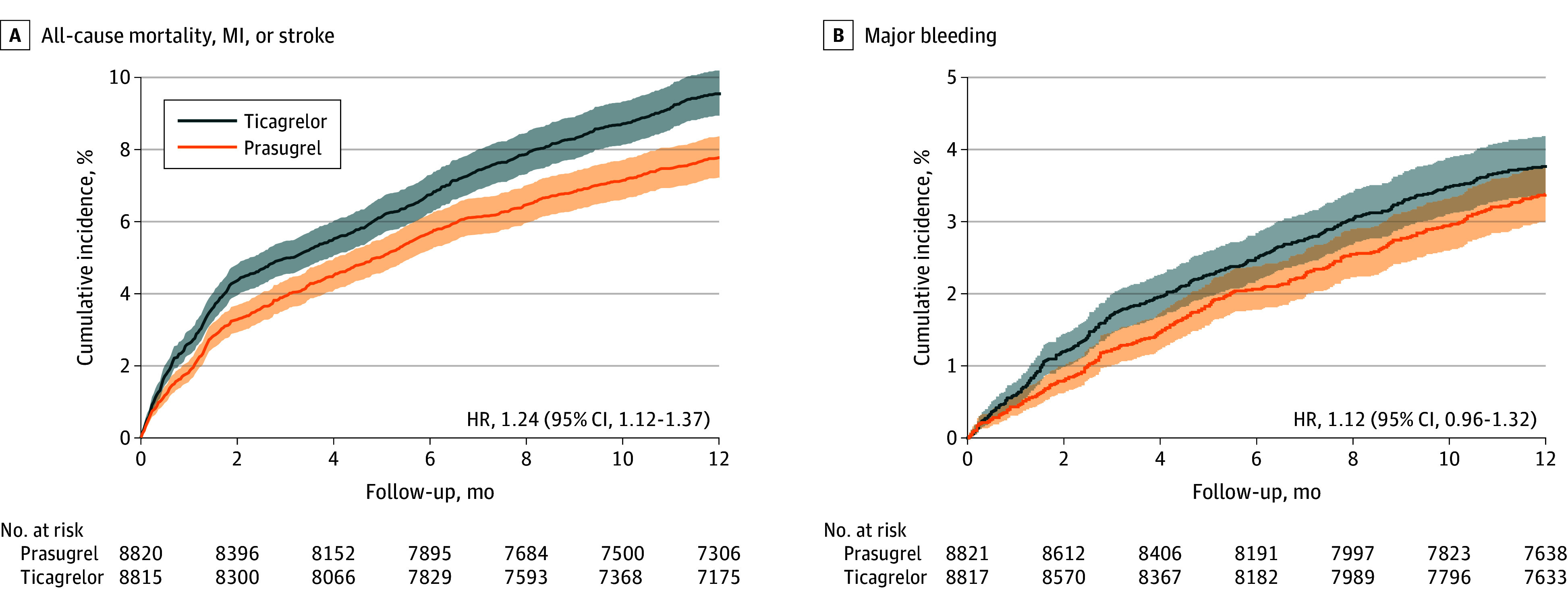
Cumulative Incidence of the Primary End Point and Safety End Point at 1 Year The Kaplan-Meier curves show the cumulative incidence of the primary composite end point (all-cause mortality, myocardial infarction [MI], or stroke) and of the safety outcome, major bleeding. Aalen-Johansen estimates are provided for bleeding considering competing risk of death.

**Figure 3.**
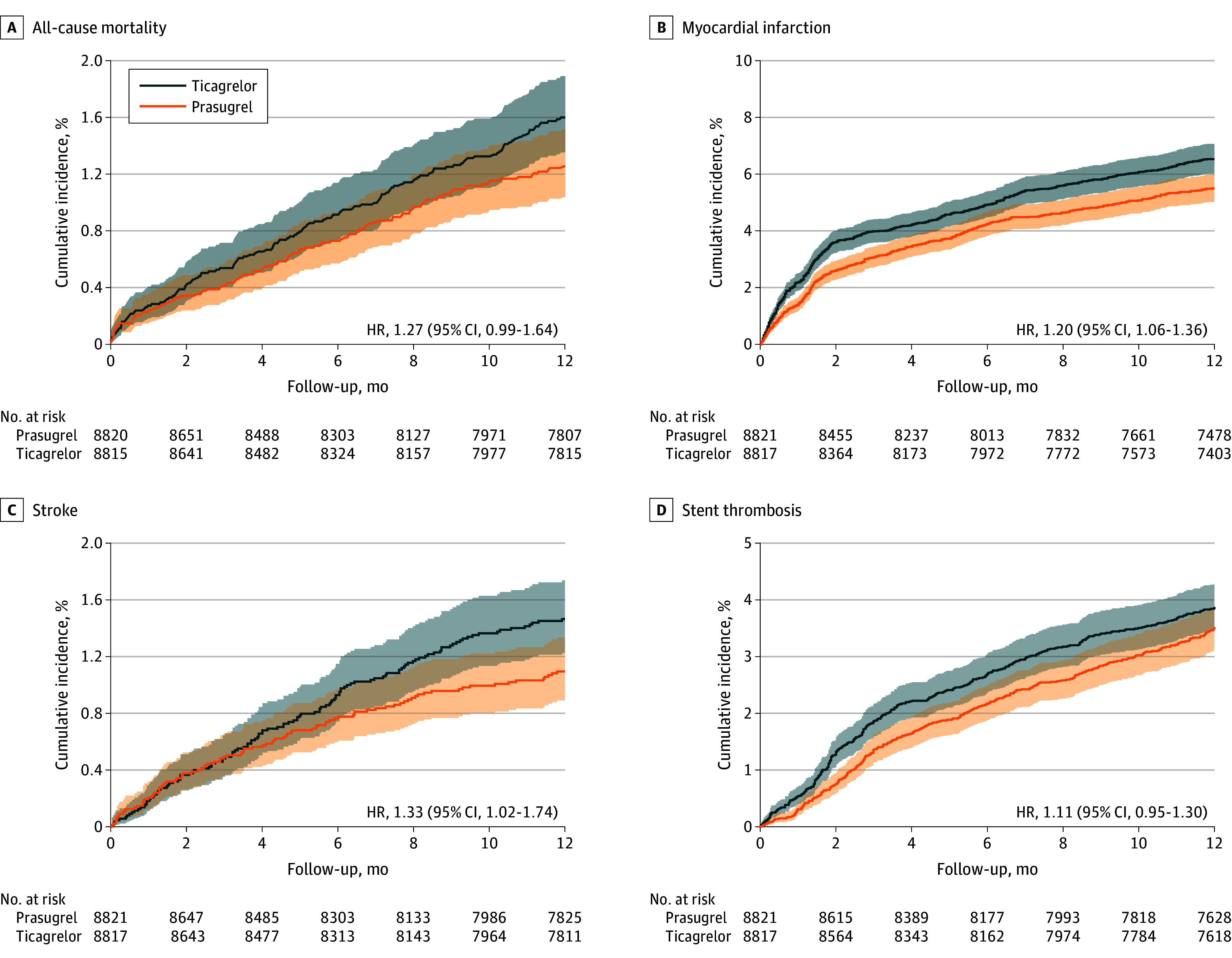
Cumulative Incidence of Secondary End Points at 1 Year The Kaplan-Meier curves show the cumulative incidence of the secondary end points—individual components of the primary end point (all-cause mortality, myocardial infarction [MI], and stroke) as well as stent thrombosis. Aalen-Johansen estimates are provided for MI, stroke, and stent thrombosis considering competing risk of death.

At 1-year follow-up, the primary end point (all-cause mortality, MI, or stroke) occurred in 815 individuals in the ticagrelor group (9.2%) vs 663 in the prasugrel group (7.5%; HR, 1.24; 95% CI, 1.12-1.37). Secondary end points in the ticagrelor vs prasugrel groups, respectively, included all-cause mortality in 135 (1.5%) vs 106 (1.2%; HR, 1.27; 95% CI, 0.99-1.64), MI in 560 (6.4%) vs 470 (5.3%; HR, 1.20; 95% CI, 1.06-1.36), stroke in 124 (1.4%) vs 93 (1.1%; HR, 1.33; 95% CI, 1.02-1.74), and stent thrombosis in 328 (3.7%) vs 295 (3.3%; HR, 1.11; 95% CI, 0.89-1.30). The safety end point of major bleeding occurred in 320 individuals in the ticagrelor group (3.6%) vs 285 in the prasugrel group (3.2%; HR, 1.12; 95% CI, 0.96-1.32) (Figure 2, Figure 3, and Table 2).

**Table 2.** Study End Points

Outcome	Participants, No. (%)		HR (95% CI)
	Ticagrelor	Prasugrel	
**Any ACS**			
Total, No.	8821	8821	NA
Primary end point^a^	815 (9.2)	663 (7.5)	1.24 (1.12-1.37)
All-cause mortality	135 (1.5)	106 (1.2)	1.27 (0.99-1.64)
Myocardial infarction	560 (6.4)	470 (5.3)	1.20 (1.06-1.36)
Stroke	124 (1.4)	93 (1.1)	1.33 (1.02-1.74)
Stent thrombosis	328 (3.7)	295 (3.3)	1.11 (0.89-1.30)
Safety end point^b^	320 (3.6)	285 (3.2)	1.12 (0.96-1.32)
**STEMI**			
Total, No.	4852	4941	NA
Primary end point^a^	451 (9.3)	338 (6.8)	1.38 (1.20-1.59)
Safety end point^b^	147 (3.0)	146 (3.0)	1.03 (0.82-1.30)
**NSTEMI**			
Total, No.	3314	3244	NA
Primary end point^a^	321 (9.7)	297 (9.2)	1.04 (0.89-1.23)
Safety end point^b^	147 (4.4)	113 (3.5)	1.26 (0.99-1.61)
**Unstable angina**			
Total, No.	655	636	NA
Primary end point^a^	43 (6.6)	28 (4.4)	1.50 (0.94-2.44)
Safety end point^b^	26 (4.0)	26 (4.1)	0.98 (0.56-1.68)

Abbreviations: ACS, acute coronary syndrome; HR, hazard ratio; NA, not applicable; NSTEMI, non-ST-segment elevation myocardial infarction; STEMI, ST-segment elevation myocardial infarction.

^a^ All-cause mortality, myocardial infarction, or stroke.

^b^ Major bleeding.

The median time interval between ACS diagnosis and the first outpatient prescription of ticagrelor or prasugrel was 7.0 days (IQR, 5.0-9.0 days; mean [SD], 7.2 [2.7] days) vs 7.0 days (IQR, 5.0-8.0 days; mean [SD], 7.0 [2.6] days). Within this period, in-hospital mortality among individuals with ACS within the insurance claims database was 1675 of 78 545 (2.1%).

The on-treatment analysis also found that ticagrelor was significantly associated with inferior outcomes for the primary end point (HR, 1.14; 95% CI, 1.03-1.28) (eTable 6 in Supplement 1). When also adjusting the analysis for age-adjusted doses (5 mg of prasugrel after age 75 years), the median on-treatment follow-up for the ticagrelor group was 11.0 months (IQR, 11.0-12.0 months; mean [SD], 10.6 [2.2] months), while for the prasugrel group, it was 12.0 months (IQR, 12.0-12.0 months; mean [SD], 11.2 [1.9] months).

Sensitivity analyses confirmed consistent results. Propensity score matching with extended preexposure characteristics (n = 71) showed an association of ticagrelor with more events related to the primary composite end point (HR, 1.25; 95% CI, 1.13-1.38). Results for the negative control outcome of influenza revealed no significant difference between the treatment groups (HR, 0.77; 95% CI, 0.45-1.32). Captured prescription fills for aspirin were comparable in both groups (4755 individuals [53.9%] for ticagrelor vs 4847 [54.9%] for prasugrel) within 14 days of ACS diagnosis.^21,22,23,24^

When comparing the results with those of IR5, the database study reached regulatory agreement for the primary composite end point, all secondary end points except stroke, and the safety end point. Concordance in estimate agreement was observed across all end points between the database evidence and IR5 results (eFigure 1 and eTable 5 in Supplement 1).

In the subgroup with STEMI, the primary end point occurred in 451 of 4852 individuals in the ticagrelor group (9.3%) vs 338 of 4941 in the prasugrel group (6.8%; HR, 1.38; 95% CI, 1.20-1.59), with comparable bleeding rates (HR, 1.03; 95% CI, 0.82-1.30) (Table 2 and eFigures 2 and 3 in Supplement 1). In the NSTEMI subgroup, the primary end point rates were 321 of 3314 (9.7%) for the ticagrelor group and 297 of 3244 (9.2%) for the prasugrel group (HR, 1.04; 95% CI, 0.89-1.23), while there was no difference in the rate of bleeding (HR, 1.26; 95% CI, 0.99-1.61) (Table 2). In the subgroup with UA, there were no statistically significant differences observed for the primary end point, which occurred in 43 of 655 individuals (6.6%) in the ticagrelor group vs 28 of 636 (4.4%) in the prasugrel group (HR, 1.50; 95% CI, 0.94-2.44). No significant difference was observed between ticagrelor and prasugrel for the safety end point (HR, 0.98; 95% CI, 0.56-1.68) in the subgroup with UA (Table 2).

## Discussion

In the setting of routine care, this cohort study found that prasugrel was associated with a significantly lower incidence of the primary composite end point (all-cause mortality, MI, or stroke) compared with ticagrelor in individuals discharged from the hospital after undergoing an invasive treatment strategy for ACS, particularly those with STEMI. In contrast, no significant difference in major bleeding between prasugrel and ticagrelor was found.

While there is no perfect emulation of an RCT with secondary data, the present study demonstrated complementary evidence for IR5^1^ after closely emulating its research question, akin to the target trial framework.^25,26^ After appropriate methods were applied, meeting 11 of 12 agreement metrics provided confidence in the validity of the results. The deviation in 1 agreement metric may have been attributable to the augmented power of the present study.

The present study had sufficient power to analyze the distinct ACS components that were underpowered in IR5.^27,28^ In individuals with STEMI, prasugrel was associated with a 38% relative risk reduction for the primary end point compared with ticagrelor, supporting use of prasugrel for STEMI, while both drugs were associated with similar risk among individuals with NSTEMI and UA. Thus, this study may have implications for the choice of P2Y12 receptor inhibitor for ACS components, particularly in patients with STEMI, as indirectly supported by data from other trials.^14,15^ In PLATO, ticagrelor compared with clopidogrel did not significantly reduce ischemic events in a predefined subgroup analysis of 7544 patients with STEMI (HR, 0.87; 95% CI, 0.75-1.01).^14^ In contrast, TRITON-TIMI 38 showed that prasugrel significantly reduced ischemic events compared with clopidogrel in a smaller cohort of 3534 patients with STEMI (HR, 0.79; 95% CI, 0.65-0.97).^15^ Though not direct head-to-head comparisons, these results also suggest that prasugrel may more effectively reduce ischemic events in STEMI, potentially due to its irreversible platelet inhibition, which is especially important in patients with STEMI undergoing an interventional strategy.^29,30^

While our results suggest a possible advantage of prasugrel for the treatment of ACS in routine care, particularly in STEMI, 2 network meta-analyses of 12^31^ and 23^32^ heterogeneous RCTs (including IR5,^1^ PRAGUE-18,^2,3^ PLATO,^14^ and TRITON-TIMI 38^15^) found no significant difference between the 2 P2Y12 receptor inhibitors, potentially due to differences in study design, populations, and follow-up.^31,32^ In contrast, another large database study showed a numerically lower event rate for prasugrel vs ticagrelor for multiple end points, including MI, at 180 days, with the effect differences becoming more pronounced over time.^33^

### Limitations

By design, this study has inherent limitations. First, we recognize important design differences between this study and IR5.^1^ Whereas randomization in IR5 took place in hospital before the invasive treatment strategy was performed, the present study omitted the in-hospital period by setting time 0 as the first observed outpatient initiation of ticagrelor or prasugrel after ACS treatment, as in-hospital medication data were not available. Consequently, early events were not captured, which may explain why we did not observe higher event rates. To address discrepancies in early event capture, we calculated in-hospital mortality in the entire cohort with ACS regardless of subsequent treatment. By adding these data to the mortality observed in the primary study cohort, we found a mortality rate comparable to those in IR5 for both ticagrelor (3.6%) and prasugrel (3.3%).^1^ Furthermore, in PRAGUE-18,^2,3^ which had its primary end point at 7 days after ACS, there was no difference in in-hospital events between both treatment groups. We could not study the role of aspirin in the comparative effectiveness of the P2Y12 receptor inhibitors, as the database used does not reliably capture over-the-counter drug use. However, captured prescription fills for aspirin were comparable in both groups, and dual-antiplatelet therapy was recommended by the European Society of Cardiology throughout the study period.^21,22,23,24^

Second, the observational nature of our study limits its ability to infer causality due to the potential of confounding. To address this, we used 1:1 propensity score matching supplemented by a sensitivity analysis on an expanded set of predefined preexposure characteristics, which affirmed the consistency of the results. Similar incidence rates in the negative control outcome of influenza further support the minimal impact of residual confounding.

Third, although both ticagrelor and prasugrel had equivalent regulatory approvals and guideline recommendations (class I, level B)^21,22,23,24^ during the study period, potential confounding by indication cannot be entirely ruled out. However, the choice between the drugs was primarily at the discretion of the treating physician, rather than based on specific indications. To further minimize potential bias, we incorporated year of ACS onset as a variable in the propensity score to adjust for temporal variations in clinical practice.

Fourth, while administrative codes offer an objective method of classifying medical concepts, their accuracy depends on coding precision and physician judgment. This may particularly affect conditions with subjective diagnostic criteria, such as UA and bleeding, but is less likely to impact conditions with clear diagnostic definitions, such as STEMI or NSTEMI, that have demonstrated high predictive power in administrative claims.^34,35^ Misclassification of outcomes is expected to be nondifferential between groups, minimizing bias in comparative analyses.

Fifth, the reliance on a German health claims database may limit the generalizability of our results to other ethnicities and health care systems. However, the database used reflects German routine care, and the Bavarian population closely approximates the Central-Western European population studied in IR5.

## Conclusions

In this cohort study of 17 642 individuals discharged after an invasive treatment strategy for ACS, prasugrel was associated with significantly lower event rates compared with ticagrelor in the primary composite outcome of all-cause mortality, MI, or stroke, without excess bleeding. These findings support the superior effectiveness of prasugrel over ticagrelor observed in IR5.^1^ The database study also allowed for robust analyses of ACS subtypes to complement underpowered RCT results. Prasugrel was associated with better outcomes compared with ticagrelor in individuals with STEMI, whereas no significant differences were observed in individuals with NSTEMI or UA. Overall, this study supports guideline recommendations preferring prasugrel over ticagrelor in patients with acute MI who are intended to undergo an invasive treatment strategy.

## Notes

Supplement 1. **eTable 1.** Diagnoses, Procedures, and Medication List **eTable 2.** Eligibility Criteria Emulation **eTable 3.** Baseline Preexposure Variables for Propensity Score Matching **eTable 4.** Extended Preexposure Variables for Propensity Score Matching **eTable 5.** Detailed Comparison of Results Between Database Study and IR5 **eTable 6.** Sensitivity Analyses **eFigure 1.** Comparison of Primary and Safety End Points Between Database Study and IR5 **eFigure 2.** STEMI Subgroup Analysis—Primary End Point **eFigure 3.** STEMI Subgroup Analysis—Safety End Point

Supplement 2.
DigiMed Bayern Consortium

Supplement 3.
Data Sharing Statement
