# Errors in the Results, Table 2, Figures 2 and 3, and Supplement 1

**DOI:** 10.1001/jamanetworkopen.2024.60952

**Published:** 2025-01-17

In the Original Investigation titled “Ticagrelor vs Prasugrel for Acute Coronary Syndrome in Routine Care,”^1^ published December 2, 2024, in the third paragraph of the Results and in Table 2, the frequencies of myocardial infarction, stroke, stent thrombosis, and major bleeding in patients with any acute coronary syndrome in the ticagrelor and prasugrel groups were updated. In Table 2, the frequency of major bleeding was also updated for patients with ST-segment elevation myocardial infarction (STEMI), non-STEMI, and unstable angina. In Figures 2 and 3, the numbers at risk were revised for both treatment groups in all panels. In eTable 5 in Supplement 1, the frequencies of myocardial infarction, stroke, stent thrombosis, and major bleeding were revised for both treatment groups. This article has been corrected.^1^ The authors have explained what happened in a Comment.^2^

## References

[1] Krüger N, Krefting J, Kessler T, ; DigiMed Bayern Consortium. Ticagrelor vs prasugrel for acute coronary syndrome in routine care. JAMA Netw Open. 2024;7(12):e2448389. doi:10.1001/jamanetworkopen.2024.48389 39621344 PMC11612834

[2] Krüger N. Comment: errors in results, tables, figures, and supplement 1. *JAMA Network Open*. December 13, 2024. https://jamanetwork.com/journals/jamanetworkopen/article-abstract/2827186

