# Supplementary material for: Ticagrelor vs Prasugrel for Acute Coronary Syndrome in Routine Care
Source: JAMA Netw Open. 2024 Dec 2;7(12):e2448389. doi: 10.1001/jamanetworkopen.2024.48389 (PMC11612834; doi:10.1001/jamanetworkopen.2024.48389)
Supplement: Supplement 1. — eTable 1. Diagnoses, Procedures, and Medication List eTable 2. Eligibility Criteria Emulation eTable 3. Baseline Preexposure Variables for Propensity Score Matching eTable 4. Extended Preexposure Variables for Propensity Score Matching eTable 5. Detailed Comparison of Results Between Database Study and IR5 eTable 6. Sensitivity Analyses eFigure 1. Comparison of Primary and Safety End Points Between Database Study and IR5 eFigure 2. STEMI Subgroup Analysis—Primary End Point eFigure 3. STEMI Subgroup Analysis—Safety End Point [file jamanetwopen-e2448389-s001.pdf]

Supplementary Online Content

Krüger N, Krefting J, Kessler T, et al; DigiMed Bayern Consortium. Ticagrelor vs prasugrel for acute coronary syndrome in routine care. *JAMA Netw Open*. 2024;7(12):e2448389. doi:10.1001/jamanetworkopen.2024.48389

eTable 1. Diagnoses, Procedures, and Medication List

eTable 2. Eligibility Criteria Emulation

eTable 3. Baseline Preexposure Variables for Propensity Score Matching

eTable 4. Extended Preexposure Variables for Propensity Score Matching

eTable 5. Detailed Comparison of Results Between Database Study and IR5

eTable 6. Sensitivity Analyses

eFigure 1. Comparison of Primary and Safety End Points Between Database Study and IR5

eFigure 2. STEMI Subgroup Analysis—Primary End Point

eFigure 3. STEMI Subgroup Analysis—Safety End Point

This supplementary material has been provided by the authors to give readers additional information about their work.

© 2024 Krüger N et al. *JAMA Network Open*.

<u>eTable 1</u> – Diagnoses, procedures and medication list. Provided are International Classification of Diseases 10th Revision (ICD-10-GM), German procedure classification system (OPS) and Anatomical Therapeutic Chemical classification system (ATC) codes used in this study.

**Diagnosis / Procedure / Medication**	**Code**
International Classification of Diseases 10th Revision (ICD-10-GM)	
Diabetes Mellitus	E10, E11, E12, E13, E14
Diabetes with Endorgan Involvement	E11.2, E11.3, E11.4, E11.5, E10.2, E10.3, E10.4, E10.5
Adipositas	E66
Dyslipidemia	E78
Aclohol Abuse	F10
Nicotine Abuse	F17
Transient Ischemic Attack	G45
Arterial Hypertension	I10, I15
Malignant Hypertension	I10.1
Hypertensive Heart and/or Kidney Disease	I11, I12, I13
Unstable Angina Pectoris	I20.0
Stable Angina Pectoris	I20.8
Acute Myocardial Infarction	I21, I22
ST- Segment Elevation Myocardial Infarction	I21.0, I21.1, I21.2, I21.3, I22.0, I22.1, I22.8
Non-ST Elevation Myocardial Infarction	I21.4
Stent Thrombosis	I25.16
Chronic Ischemic Heart Disease	I25
Chronic Ischemic Heart Disease with at least 2 vessel disease	I25.12, I25.13
Atherosclerotic Heart Disease	I25.1
Old Myocardial Infarction	I25.2
Pulmonary Embolism	I26
AV Block Grade II and III	I44.1, I44.2
Other Cardiac Arrhythmias	I49
Atrial Fibrilation and Flutter	I48
Sick Sinus Syndrome	I49.5
Chronic Heart Failure	I50
Intracranial Hemorrhage	I61

© 2024 Krüger N et al. *JAMA Network Open*.

Major Bleeding	I60, I61, I62, K92.0, K92.1, K92.2
Acute Stroke	I63, I64
Intracranial Arteriovenous Malformation or Intracranial Aneurysm	I67.1
Atherosclerosis	I70
Aortic Atherosclerosis	I70.0
Renal Atherosclerosis	I70.1
Carotid stenosis	I65.2
Peripheral Artery Disease	I70.2
Arterial Embolism Or Thrombosis	I74
Venous Thrombosis	I80, I81, I82
Pneumonia	J13, J14, J15, J16
COPD	J44
Asthma	J45
Acute Kidney Failure	N17
Chronic Kidney Disease	N18*
Chronic Kidney Failure Stage 1 And 2	N18.1, N18.2
Chronic Kidney Failure Stage 3, 4 And 5	N18.3, N18.4, N18.5
Chronic Renal Insufficiency Requiring Dialysis	N18.5
Other Chronic Kidney Disease	N18.8, N18.9
German procedure classification system (OPS)	
Diagnostic Cardiac Catheterization	1-275*
(Percutaneous) Transluminal Stenting	8-837* Excl.: 8-837.7*, 8-837.8, 8-837.9, 8-837.a*, 8-837.b*, 8-837.c*, 8-837.d*, 8-837.e, 8-837.f, 8-837.g, 8-837.h, 8-837.j, 8-837.s*
Coronary Artery Bypass Graft Surgery	5-36*
Complex treatment	8-98*
Anatomical Therapeutic Chemical classification system (ATC)	
Heparin	B01AB
Antiarrhythmics	C01B, C01AA
Nitrates	C01DA
Antihypertensives	C02

© 2024 Krüger N et al. *JAMA Network Open*.

Thiazides	C03A, C03EA, C07B, C07D, C09BA21, C09BA22, C09BA23, C09BA25, C09BA26, C09BA27, C09BA28, C09BA29, C09BA33, C09BA35, C09BA54, C09DA21, C09DA22, C09DA23, C09DA24, C09DA26, C09DA27, C09DA28
Other Diuretics	C03B, C03DB, C03EA, C03EB, C03EC, C03X, C09BA01, C09BA02, C09BA03, C09BA04, C09BA05, C09BA06, C09BA07, C09BA08, C09BA09, C09BA12, C09BA13, C09BA15, C09DA01, C09DA02, C09DA03, C09DA04, C09DA06, C09DA07, C09DA08, C09DA09, C09DA10
Loop Diuretics	C03C, C03EB, C03ED, C07C, C07D, C09BA55
Aldosteron Antagonists	C03DA, C03EC, C03ED
Beta Blocking Agents	C07A, C07B, C07C, C07D, C07E, C07FB
Calcium Channel Blockers	C07FB, C08, C09BB, C09DB
ACE Inhibitors	C09A, C09BA01, C09BA02, C09BA03, C09BA04, C09BA05, C09BA06, C09BA07, C09BA08, C09BA09, C09BA12, C09BA13, C09BA15, C09BA21, C09BA22, C09BA23, C09BA25, C09BA26, C09BA27, C09BA28, C09BA29, C09BA33, C09BA35, C09BA54, C09BA55, C09BB
Angiotensin II Receptor Blockers	C09CA, C09DA01, C09DA02, C09DA03, C09DA04, C09DA06, C09DA07, C09DA08, C09DA09, C09DA10, C09DA21, C09DA22, C09DA23, C09DA24, C09DA26, C09DA27, C09DA28, C09DB

© 2024 Krüger N et al. *JAMA Network Open*.

Angiogenesis Inhibitors	C09DX
Statins	C10AA
Other Lipid Modifying Agents	C10AB, C10AC, C10AD, C10AX
Oral Immunosuppressants	L04
NSAIDs	M01A, M01BA, N02BA
Opioids	N02A
Non-Opioid Analgetica	N02BB, N02BE, N02BG
Drugs for obstructive airway diseases	R03

© 2024 Krüger N et al. *JAMA Network Open*.

eTable 2 – Elegibility criteria emulation. Provided are the inclusion and exclusion criteria of IR5 and their applicability in the database study. 1. The combination of diagnosis and invasive strategy is used to emulate "planned invasive strategy" 2. Inherent in the coding of the associated ICD-10-GM diagnosis. 3. Only 1 of the following criteria was required to apply for this emulation

Inclusion Criteria IR5	Applicability in database study
Hospitalization for an Acute Coronary Syndrome: STEMI, NSTEMI or Unstable Angina Pectoris<br>● With Planned Invasive Strategy	Yes<br>Indirectly [1]
ST-Segment Elevation myocardial Infarction	Yes
● Chest discomfort suggestive of cardiac ischemia ≥ 20 minutes at rest, within 24 h prior to randomization	No
● 1 of the following ECG features:<br>○ ST-segment elevation ≥ 1 mm in ≥ 2 contiguous ECG leads or<br>○ new or presumably new left bundle branch block (LBBB)	Indirectly [2]
Non-ST-Segment Elevation myocardial Infarction	Yes
● Chest discomfort suggestive of cardiac ischemia for ≥ 10 minutes at rest within 48 h prior to randomization	Indirectly [2]
● And 1 of the following criteria:<br>○ ST-segment depression ≥ 1 mm in ≥ 1 or 2 contiguous ECG leads or<br>○ Troponin T or I or CK-MB greater than the upper limit of normal	Indirectly [2]
● Or 2 of the following clinical criteria:<br>○ Age ≥ 60 years<br>○ ≥ 3 risk factors for coronary artery disease:<br>arterial hypertension,<br>hypercholesterolemia,<br>family history,<br>diabetes mellitus,<br>current smoker<br>○ Diabetes mellitus<br>○ Aspirin use in the past 7 days<br>○ Severe angina (≥ 2 episodes within the last 24 hours)<br>○ Chronic renal dysfunction<br>○ Prior MI or CABG	[3]<br>Yes<br><br>Yes<br>Yes<br>No<br>Yes<br>Yes<br>Yes<br>No<br>Indirectly [2]<br>Yes<br>Yes

© 2024 Krüger N et al. *JAMA Network Open*.

○ Known CAD with ≥ 50% stenosis in ≥ 2 vessels<br>○ Carotid stenosis ≥ 50% or cerebral revascularization<br>○ Peripheral artery disease	Indirectly [2]<br>Indirectly [2]<br>Yes
Age ≥18 Years	Yes
**Exclusion Criteria IR5**	**Applicability in database study**
Intolerance of/ or allergy to Ticagrelor or Prasugrel	Indirectly [2]
History of any stroke, transient ischemic attack, or intracranial bleeding	Yes
Known intracranial neoplasm	No
Intracranial arteriovenous malformation or intracranial Aneurysm	Yes
Fibrin-specific fibrinolytic therapy less than 24 h before randomization, non-fibrinspecific fibrinolytic therapy less than 48h before randomization	No
Known platelet count < 100.000/ml at the time of screening	No
Clinical findings, that in the judgment of the investigator are associated with an increased risk of bleeding	No
Known anemia (Hemoglobin < 10 g/dl) at the time of screening	No
Oral anticoagulation	Yes
INR known to be greater than 1.5 at the time of screening	No
Chronic renal insufficiency requiring dialysis	Yes
Moderate or severe hepatic dysfunction (Child Pugh B or C)	No
Increased risk of bradycardia events (Sick Sinus, AV block grade II or III, Bradycardia-Induced Syncope)	Yes
Index event is an acute complication (< 30 days) of PCI	No
Concomitant medical illness that in the opinion of the investigator is associated with a life expectancy < 1 year	No
Concomitant oral or i.v. Therapy with strong CYP3A inhibitors (e.g. Ketoconazole, Itraconazole, Voriconazole, Telithromycin, Clarithromycin, Nefazodone, Ritonavir, Saquinavir, Nelfinavir, Indinavir, Atazanavir, grapefruit juice >1 l/d), CYP3A substrates with narrow therapeutic indices (e.g. Cyclosporine, Quinidine), or strong CYP3A inducers (e.g. Rifampin/Rifampicin, Phenytoin, Carbamazepine, Dexamethason, Phenobarbital) that cannot be safely discontinued	No
≥ 1 doses of ticagrelor or prasugrel within 5 days before randomization	Yes
Pregnancy, giving birth within the last 90 days, or lactation	No
Inability to cooperate with protocol requirements	No

<u>eTable 3</u> – Baseline preexposure variables for propensity score matching. Provided are the individual variables for the applied baseline propensity score matching used in the study.

1	Age
2	Sex
3	Chronic Heart Failure
4	Nicotine Abuse
5	Diabetes Mellitus
6	Chronic Kidney Disease
7	Obesity
8	Arterial Hypertension
9	Hyperlipidemia
10	Chronic Ischemic Heart Disease
11	Peripheral Arterial Disease
12	ST-Elevation Myocardial Infarction
13	Non-ST-Elevation Myocardial Infarction
14	Unstable Angina Pectoris
15	Cardiac Catheterization
16	Percutaneous Coronary Intervention within the last year
17	Coronary Arotic Bypass Grafting within the last year
18	Insulin intake
19	ACS hospitalization in 2013
20	ACS hospitalization in 2014
21	ACS hospitalization in 2015
22	ACS hospitalization in 2016
23	ACS hospitalization in 2017
24	ACS hospitalization in 2018
25	ACS hospitalization in 2019
26	ACS hospitalization in 2020
27	ACS hospitalization in 2021

© 2024 Krüger N et al. *JAMA Network Open*.

eTable 4 – Extended preexposure variables for propensity score matching. Provided are the individual variables for the applied extended propensity score matching used in the study.

1	Age
2	Sex
3	Nicotine Abuse
4	Alcohol Abuse
5	Hypertensive Heart and/or Kidney Disease
6	Acute Kidney Disease
7	Chronic Kidney Disease Stage 1 or 2
8	Chronic Kidney Disease Stage 3, 4, or 5 incl. Dialysis
9	Chronic Kidney Disease not Further Described
10	Diabetes Mellitus
11	Diabetes Mellitus with End Organ Involvement
12	Obesity
13	Arterial Hypertension
14	Malignant Hypertension
15	Hyperlipidemia
16	Atrial Fibrillation
17	Other Cardiac Arrythmias
18	Stable Angina
19	Chronic Ischemic Heart Disease
20	Old Myocardial Infarction
21	Chronic Heart Failure
22	Peripheral Artery Disease
23	Arterial Embolism or Thrombosis
24	Thrombosis
25	Lung Embolism
26	Pneumonia
27	Chronic Obstructive Pulmonary Disease
28	Bronchial Asthma
29	History Of Percutaneous Coronary Intervention
30	History Of Coronary Aortic Bypass Graft
31	Medical Procedure Count
32	Intensive Care Count
33	ST-Elevation Myocardial Infarction
34	Non-ST-Elevation Myocardial Infarction

© 2024 Krüger N et al. *JAMA Network Open*.

35	Unstable Angina Pectoris
36	Diagnostic Heart Catheter
37	Percutaneous Coronary Intervention within the last year
38	Coronary Arotic Bypass Grafting within the last year
39	Unique Medication Count
40	Ace Inhibitors
41	Angiotensin receptor blockers
42	Beta Blocking Agents
43	Calcium Channel Blockers
44	Insulin
45	SGLT2 Inhibitors
46	Other Antidiabetic Medications
47	Antiplatelet Agents
48	Heparin
49	Anticoagulants
50	Nonsteroidal Anti-inflammatory Drugs
51	Non Opioid Analgesics
52	Opioids
53	Antiarrhythmics
54	Antihypertensives
55	Loop Diuretics
56	Thiazide
57	Other Diuretics
58	Aldosterone Antagonists
59	Statins
60	Other Lipid Modifying Agents
61	Anti-Obstructive Medication
62	Charlson Comorbidity Index from Year Before
63	ACS hospitalization in 2013
64	ACS hospitalization in 2014
65	ACS hospitalization in 2015
66	ACS hospitalization in 2016
67	ACS hospitalization in 2017
68	ACS hospitalization in 2018
69	ACS hospitalization in 2019
70	ACS hospitalization in 2020

© 2024 Krüger N et al. *JAMA Network Open*.

71	ACS hospitalization in 2021

© 2024 Krüger N et al. *JAMA Network Open*.

eTable 5 – Detailed comparison of results between database study and IR5. End points provided for the main analysis. Values are provided as n (%). MI, myocardial infarction.

End points database study	Ticagrelor Group (n=8,821)	Prasugrel Group (n=8,821)	Hazard Ratio (95% CI)
Primary end point: all-cause mortality, MI, or stroke	**815 (9.2%)**	**663 (7.5%)**	**1.24 (1.12-1.37)**
All-cause mortality	135 (1.5%)	106 (1.2%)	1.27 (0.99-1.64)
MI	**560 (6.4%)**	**470 (5.3%)**	**1.20 (1.06-1.36)**
Stroke	**124 (1.4%)**	**93 (1.1%)**	**1.33 (1.02-1.74)**
Stent thrombosis	328 (3.7%)	295 (3.3%)	1.11 (0.89-1.30)
Safety end point: major bleeding	320 (3.6%)	285 (3.2%)	1.12 (0.96-1.32)
**End points IR5**	**Ticagrelor Group (n=2,012)**	**Prasugrel Group (n=2,006)**	**Hazard Ratio (95% CI)**
Primary end point: all-cause mortality, MI, or stroke	**184 (9.3%)**	**137 (6.9%)**	**1.36 (1.09-1.70)**
All-cause mortality	90 (4.5%)	73 (3.7%)	1.23 (0.91-1.68)
MI	**96 (4.8%)**	**60 (3.0%)**	**1.63 (1.18-2.25)**
Stroke	22 (1.1%)	19 (1.0%)	1.17 (0.63-2.15)
Stent thrombosis	26 (1.3%)	20 (1.0%)	1.30 (0.72-2.33)
Safety end point: major bleeding	95/1,989 (5.4%)	80/1,773 (4.8%)	1.12 (0.83-1.51)

© 2024 Krüger N et al. *JAMA Network Open*.

<u>eTable 6</u> – Sensitivity analyses. Values are provided as n (%). MI, myocardial infarction; PSM, propensity score matching.

**Sensitivity analysis with extended list of preexposure variables for PSM**	**Ticagrelor Group (n=8,751)**	**Prasugrel Group (n=8,751)**	**Hazard Ratio (95% CI)**
**Primary end point: all-cause mortality, MI, or stroke**	**795 (9.1%)**	**643 (7.4%)**	**1.25 (1.13-1.38)**
**Sensitivity analysis with "on-treatment" design**	**Ticagrelor Group (n=8,821)**	**Prasugrel Group (n=8,821)**	**Hazard Ratio (95% CI)**
**Primary end point: all-cause mortality, MI, or stroke**	**681 (7.7%)**	**599 (6.8%)**	**1.14 (1.03-1.28)**

© 2024 Krüger N et al. *JAMA Network Open*.

<u>eFigure 1</u> – Comparison of primary and safety end points between database study and IR5. Primary composite end point (all-cause mortality, MI and stroke) and safety end point (major bleeding). MI, myocardial infarction.

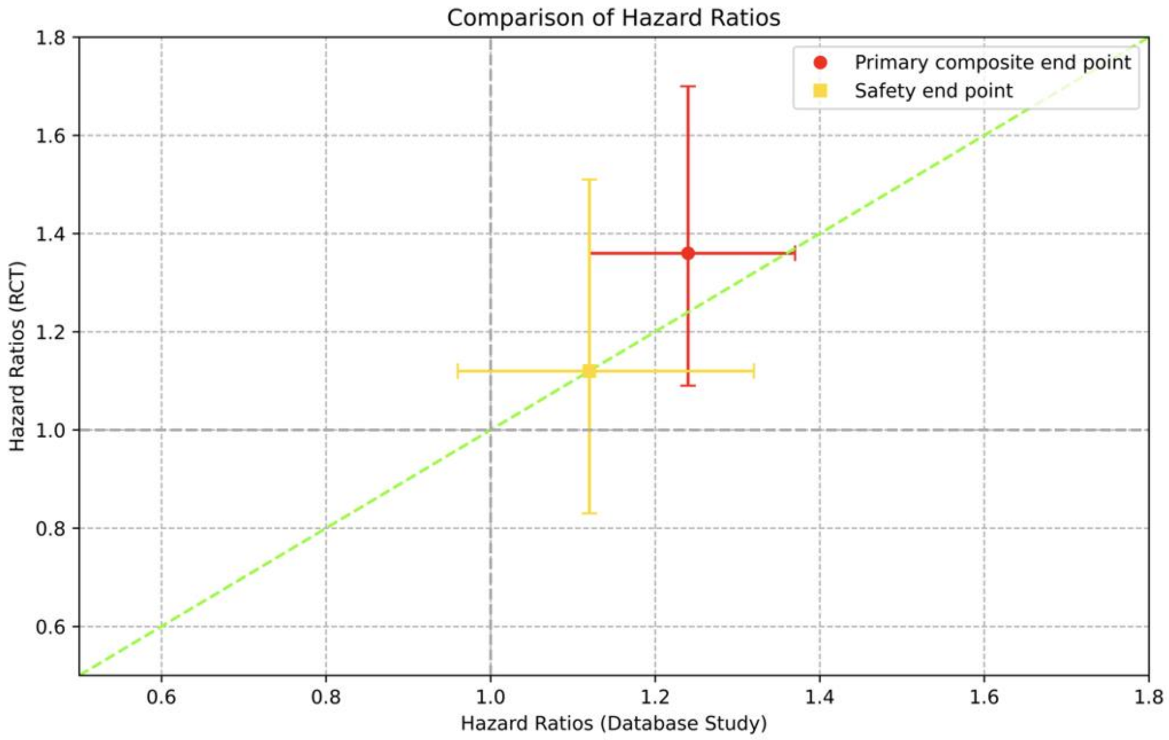

© 2024 Krüger N et al. *JAMA Network Open*.

<u>eFigure 2</u> – STEMI subgroup analysis – primary end point. The Kaplan-Meier curves show the cumulative incidence of the primary compsite end point (all-cause mortality, MI, or stroke) at 1 year. ST-segment elevation myocardial infarction; MI, myocardial infarction.

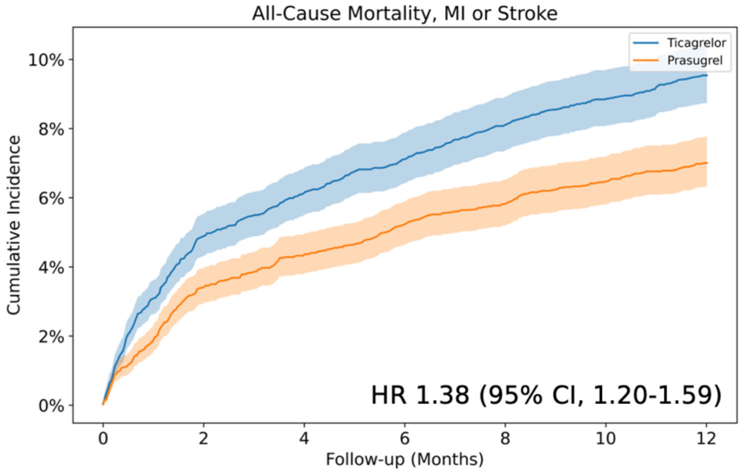

**Prasugrel**							
At risk	4940	4720	4616	4509	4409	4334	4240
Censored	0	54	113	177	249	294	363
Events	1	167	212	255	283	313	338
**Ticagrelor**							
At risk	4850	4546	4423	4313	4206	4097	4008
Censored	0	71	135	199	260	335	393
Events	2	235	294	340	386	420	451

© 2024 Krüger N et al. *JAMA Network Open*.

<u>eFigure 3</u> – STEMI subgroup analysis – safety end point. The Kaplan-Meier curves show the cumulative incidence of the safety end point major bleeding at 1 year. Aalen-Johansen estimates are provided for bleeding considering competing risk of death. ST-segment elevation myocardial infarction

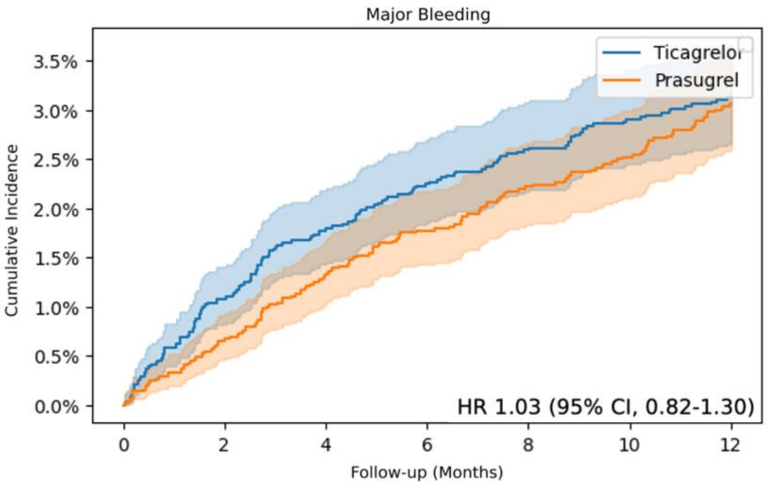

Prasugrel							
At risk	4941	4854	4760	4669	4574	4508	4412
Censored	0	55	117	186	260	312	383
Events	0	32	64	86	107	121	146
Ticagrelor							
At risk	4850	4726	4625	4538	4458	4368	4295
Censored	2	74	142	207	271	347	410
Events	0	52	85	107	123	137	147

© 2024 Krüger N et al. *JAMA Network Open*.
