# Supplementary material for: Ticagrelor vs Prasugrel for Acute Coronary Syndrome in Routine Care
Source: JAMA Netw Open. 2024 Dec 2;7(12):e2448389. doi: 10.1001/jamanetworkopen.2024.48389 (PMC11612834; doi:10.1001/jamanetworkopen.2024.48389)
Supplement: Supplement 2. — DigiMed Bayern Consortium [file jamanetwopen-e2448389-s002.pdf]

Supplemental Online Content: Groupauthor Collaborators
*First name, last name, and suffix (if applicable) are required and will appear in PubMed.

*Group Name(s): DigiMed Bayern Consortium							
***First Name and Middle Initial(s)**	***Last Name**	***Suffix (eg, Jr, III)**	Academic Degrees	Institution	Location (city, state/province, country)	Role or Contribution, eg, chair, principal investigator	Group (if more than 1 Group listed in the byline) and/or Subgroup (eg, Steering Committee)
Jonathan	Adam		PhD	Institut für Medizinische Informationsverarbeitung Biometrie und Epidemiologie, Ludwig-Maximilians-Universität München, Munich, Germany; Institut für Epidemiologie, Helmholtz Zentrum München, Munich, Germany	Munich, Bavaria, Germany	Collaborator	
Reiner	Anselm		PhD	Institut Technik – Theologie – Naturwissenschaften, Ludwig-Maximilians-Universität München, Munich, Germany	Munich, Bavaria, Germany	PI	
Sara	Ates		MD	Deutsches Herzzentrum München, Klinik für Herz- und Kreislauferkrankungen, Technische Universität München, Munich, Germany	Munich, Bavaria, Germany	Collaborator	
Sabine	Bauer		MSc	Sabine Bauer, Deutsches Herzzentrum München, Klinik für Herz- und Kreislauferkrankungen, Technische Universität München, Munich, Germany	Munich, Bavaria, Germany	Collaborator	
Nicole	Beck			Nicole Beck, Deutsches Herzzentrum München, Klinik für Herz- und Gefäßchirurgie, Technische Universität München, Munich, Germany	Munich, Bavaria, Germany	Collaborator	
Jürgen	Beckmann		PhD	Fakultät für Sport- und Gesundheitswissenschaften, Technische Universität München, Munich, Germany	Munich, Bavaria, Germany	Co-PI	

*First name, last name, and suffix (if applicable) are required and will appear in PubMed.

***First Name and Middle Initial(s)**	***Last Name**	***Suffix (eg, Jr, III)**	Academic Degrees	Institution	Location (city, state/province, country)	Role or Contribution, eg, chair, principal investigator	Group (if more than 1 Group listed in the byline) and/or Subgroup (eg, Steering Committee)
Riccardo	Berutti		PhD	Institut für Neurogenomik, Helmholtz Zentrum München, Munich, Germany	Munich, Bavaria, Germany	Collaborator	
Stefan	Brandmaier		PhD	Institut für Epidemiologie, Helmholtz Zentrum München, Munich, Germany; Theresa Brunet, Institut für Humangenetik, Technische Universität München, Munich, Germany	Munich, Bavaria, Germany	Collaborator	
Tim	Bruun		PhD	BioM Biotech Cluster Development GmbH, Martinsried, Germany	Martinsried, Bavaria, German	Collaborator	
Salvatore	Cassese		PhD	Deutsches Herzzentrum München, Klinik für Herz- und Kreislauferkrankungen, Technische Universität München, Munich, Germany	Munich, Bavaria, Germany	Collaborator	
Manuela	Decker		MSc	Deutsches Herzzentrum München, Klinik für Herz- und Kreislauferkrankungen, Technische Universität München, Munich, Germany	Munich, Bavaria, Germany	Collaborator	
Martin	Dichgans		MD	Institut für Schlaganfall- und Demenzforschung, Ludwig-Maximilians-Universität München, Munich, Germany	Munich, Bavaria, Germany	Co-PI	
Philine	Diesselhorst			Institut Technik – Theologie – Naturwissenschaften, Ludwig-Maximilians-Universität	Munich, Bavaria, Germany	Collaborator	
Horst	Domdey		PhD	BioM Biotech Cluster Development GmbH, Martinsried, Germany	Munich, Bavaria, Germany	Co-PI	

Supplemental Online Content: Groupauthor Collaborators
*First name, last name, and suffix (if applicable) are required and will appear in PubMed.

***First Name and Middle Initial(s)**	***Last Name**	***Suffix (eg, Jr, III)**	Academic Degrees	Institution	Location (city, state/province, country)	Role or Contribution, eg, chair, principal investigator	Group (if more than 1 Group listed in the byline) and/or Subgroup (eg, Steering Committee)
Stefanie	Doppler		PhD	Deutsches Herzzentrum München, Klinik für Herz- und Gefäßchirurgie, Technische Universität München, Munich, Germany	Munich, Bavaria, Germany	Collaborator	
Martina	Dreßen		PhD	Deutsches Herzzentrum München, Klinik für Herz- und Gefäßchirurgie, Technische Universität München, Munich, Germany	Munich, Bavaria, Germany	Collaborator	
Arne	Dressler		PhD	Institut Technik – Theologie – Naturwissenschaften, Ludwig-Maximilians-Universität München, Munich, Germany	Munich, Bavaria, Germany	Collaborator	
Florent	Dufour		MSc	Leibniz-Rechenzentrum, Munich, Germany	Munich, Bavaria, Germany	Collaborator	
Sven	Duscha		PhD	Deutsches Herzzentrum München, Klinik für Herz- und Kreislauferkrankungen, Technische Universität München, Munich, Germany	Munich, Bavaria, Germany	Collaborator	
Hans H.	Eckstein		MD	Klinik und Poliklinik für Vaskuläre und Endovaskuläre Chirurgie, Klinikums rechts der Isar, Technische Universität München, Munich, Germany	Munich, Bavaria, Germany	Collaborator	
Aiman	Farzeen		MSc	Institut für Humangenetik, Technische Universität München, Munich, Germany; Institut für Epidemiologie, Helmholtz Zentrum München, Munich, Germany	Munich, Bavaria, Germany	Collaborator	

Supplemental Online Content: Groupauthor Collaborators
*First name, last name, and suffix (if applicable) are required and will appear in PubMed.

***First Name and Middle Initial(s)**	***Last Name**	***Suffix (eg, Jr, III)**	Academic Degrees	Institution	Location (city, state/province, country)	Role or Contribution, eg, chair, principal investigator	Group (if more than 1 Group listed in the byline) and/or Subgroup (eg, Steering Committee)
Therese	Feiler		PhD	Institut Technik – Theologie – Naturwissenschaften, Ludwig-Maximilians-Universität München, Munich, Germany	Munich, Bavaria, Germany	Collaborator	
Christian	Friess		MD	Deutsches Herzzentrum München, Klinik für Herz- und Kreislauferkrankungen, Technische Universität München, Munich, Germany	Munich, Bavaria, Germany	Collaborator	
Ines	Gall			Deutsches Herzzentrum München, Institut für Laboratoriumsmedizin, Technische Universität München, Munich, Germany	Munich, Bavaria, Germany	Collaborator	
Ulrich M	Gassner		PhD	Juristische Fakultät, Universität Augsburg, Augsburg, Germany	Augsburg, Bavaria, Germany	PI	
Christian	Gieger		PhD	Institut für Epidemiologie, Helmholtz Zentrum München, Munich, Germany	Munich, Bavaria, Germany	Collaborator	
Monica	Gotor-Blazquez			Deutsches Herzzentrum München, Klinik für Herz- und Kreislauferkrankungen, Technische Universität München, Munich, Germany	Munich, Bavaria, Germany	Collaborator	
Ulrich	Gueldener		PhD	Deutsches Herzzentrum München, Klinik für Herz- und Kreislauferkrankungen, Technische Universität München, Munich, Germany	Munich, Bavaria, Germany	Collaborator	
Nicolay	Hammer		MSc	Max-Planck-Institut für Biochemie, Munich, Germany	Munich, Bavaria, Germany	Collaborator	

Supplemental Online Content: Groupauthor Collaborators
*First name, last name, and suffix (if applicable) are required and will appear in PubMed.

***First Name and Middle Initial(s)**	***Last Name**	***Suffix (eg, Jr, III)**	Academic Degrees	Institution	Location (city, state/province, country)	Role or Contribution, eg, chair, principal investigator	Group (if more than 1 Group listed in the byline) and/or Subgroup (eg, Steering Committee)
Johann	Hawe		PhD	Deutsches Herzzentrum München, Klinik für Herz- und Kreislauferkrankungen, Technische Universität München, Munich, Germany	Munich, Bavaria, Germany	Collaborator	
Verena	Heidel			Deutsches Herzzentrum München, Klinik für Herz- und Kreislauferkrankungen, Technische Universität München, Munich, Germany	Munich, Bavaria, Germany	Collaborator	
Thomas	Hendel			Institut für Epidemiologie, Helmholtz Zentrum München, Munich, Germany	Munich, Bavaria, Germany	Collaborator	
Stefan	Holdenrieder		MD	Deutsches Herzzentrum München, Institut für Laboratoriumsmedizin, Technische Universität München, Munich, Germany	Munich, Bavaria, Germany	Collaborator	
Stephan	Jonas		PhD	Institut für Informatik, Technische Universität München, Munich, Germany	Munich, Bavaria, Germany	PI	
Muamer	Kameric			Deutsches Herzzentrum München, Klinik für Herz- und Gefäßchirurgie, Technische Universität München, Munich, Germany	Munich, Bavaria, Germany	Collaborator	
Adnan	Kastrati		MD	Deutsches Herzzentrum München, Klinik für Herz- und Kreislauferkrankungen, Technische Universität München, Munich, Germany	Munich, Bavaria, Germany	Collaborator	

Supplemental Online Content: Groupauthor Collaborators
*First name, last name, and suffix (if applicable) are required and will appear in PubMed.

***First Name and Middle Initial(s)**	***Last Name**	***Suffix (eg, Jr, III)**	Academic Degrees	Institution	Location (city, state/province, country)	Role or Contribution, eg, chair, principal investigator	Group (if more than 1 Group listed in the byline) and/or Subgroup (eg, Steering Committee)
Thorsten	Kessler		MD	Deutsches Herzzentrum München, Klinik für Herz- und Kreislauferkrankungen, Technische Universität München, Munich, Germany	Munich, Bavaria, Germany	Collaborator	
Katharina	Knoedlseder			Deutsches Herzzentrum München, Klinik für Herz- und Kreislauferkrankungen, Technische Universität München, Munich, Germany	Munich, Bavaria, Germany	Collaborator	
Wolfgang	Koenig		MD	Deutsches Herzzentrum München, Klinik für Herz- und Kreislauferkrankungen, Technische Universität München, Munich, Germany	Munich, Bavaria, Germany	Co-PI	
Florian	Kohlmayer		PhD	Bitcare, Munich, Germany	Munich, Bavaria, Germany	Collaborator	
Markus	Krane		MD	Deutsches Herzzentrum München, Klinik für Herz- und Gefäßchirurgie, Technische Universität München, Munich, Germany	Munich, Bavaria, Germany	Collaborator	
Dieter	Kranzelmuelle r		PhD	Institut für Informatik, Ludwig-Maximilians-Universität München, Munich, Germany	Munich, Bavaria, Germany	PI	
Johannes	Krefting		MD	Deutsches Herzzentrum München, Klinik für Herz- und Kreislauferkrankungen, Technische Universität München, Munich, Germany	Munich, Bavaria, Germany	Collaborator	
Nils	Krüger		MD	Deutsches Herzzentrum München, Klinik für Herz- und Kreislauferkrankungen, Technische Universität München, Munich, Germany	Munich, Bavaria, Germany	Collaborator	

*First name, last name, and suffix (if applicable) are required and will appear in PubMed.

***First Name and Middle Initial(s)**	***Last Name**	***Suffix (eg, Jr, III)**	Academic Degrees	Institution	Location (city, state/province, country)	Role or Contribution, eg, chair, principal investigator	Group (if more than 1 Group listed in the byline) and/or Subgroup (eg, Steering Committee)
Anja	Kroke			BioM Biotech Cluster Development GmbH, Martinsried, Germany	Martinsried, Bavaria, German	Collaborator	
Harald	Lahm		PhD	Deutsches Herzzentrum München, Klinik für Herz- und Gefäßchirurgie, Technische Universität München, Munich, Germany	Munich, Bavaria, Germany	Collaborator	
Ruediger	Lange		MD	Deutsches Herzzentrum München, Klinik für Herz- und Gefäßchirurgie, Technische Universität München, Munich, Germany	Munich, Bavaria, Germany	Collaborator	
Andreas	Lehmann		MSc	Bitcare GmbH, Munich, Germany	Munich, Bavaria, Germany	Collaborator	
Ling	Li		PhD	Deutsches Herzzentrum München, Klinik für Herz- und Kreislauferkrankungen, Technische Universität München, Munich, Germany	Munich, Bavaria, Germany	Collaborator	
Birgit	Linkohr		PhD	Institut für Epidemiologie, Helmholtz Zentrum München, Munich, Germany	Munich, Bavaria, Germany	Collaborator	
Lars	Maegdefessel		MD	Klinik und Poliklinik für Vaskuläre und Endovaskuläre Chirurgie, Klinikums rechts der Isar, Technische Universität München, Munich, Germany	Munich, Bavaria, Germany	PI	
Matthias	Mann		PhD	Max-Planck-Institut für Biochemie, Munich, Germany	Munich, Bavaria, Germany	PI	
Rainer	Malik		PhD	Institut für Schlaganfall- und Demenzforschung, Ludwig-Maximilians-Universität München, Munich, Germany	Munich, Bavaria, Germany	Co-PI	

*First name, last name, and suffix (if applicable) are required and will appear in PubMed.

***First Name and Middle Initial(s)**	***Last Name**	***Suffix (eg, Jr, III)**	Academic Degrees	Institution	Location (city, state/province, country)	Role or Contribution, eg, chair, principal investigator	Group (if more than 1 Group listed in the byline) and/or Subgroup (eg, Steering Committee)
Thomas	Meitinger		PhD	Institut für Humangenetik, Technische Universität München, Munich, Germany	Munich, Bavaria, Germany	Co-PI	
Irina	Neb			Deutsches Herzzentrum München, Klinik für Herz- und Gefäßchirurgie, Technische Universität München, Munich, Germany	Munich, Bavaria, Germany	Collaborator	
Tina	O'Hehir			Deutsches Herzzentrum München, Klinik für Herz- und Kreislauferkrankungen, Technische Universität München, Munich, Germany	Munich, Bavaria, Germany	Collaborator	
Shichao	Pang		PhD	Deutsches Herzzentrum München, Klinik für Herz- und Kreislauferkrankungen, Technische Universität München, Munich, Germany	Munich, Bavaria, Germany	Collaborator	
Benedikt	Perl		MSc	Fakultät für Sport- und Gesundheitswissenschaften, Technische Universität München, Munich, Germany	Munich, Bavaria, Germany	Collaborator	
Annette	Peters		PhD	Institut für Medizinische Informationsverarbeitung Biometrie und Epidemiologie Ludwig-Maximilians-Universität München, Munich, Germany; Institut für Epidemiologie, Helmholtz Zentrum München, Munich, Germany	Munich, Bavaria, Germany	PI	
Fatemeh	Peymani		MSc	Institut für Humangenetik, Technische Universität München, Munich, Germany	Munich, Bavaria, Germany	Collaborator	

*First name, last name, and suffix (if applicable) are required and will appear in PubMed.

***First Name and Middle Initial(s)**	***Last Name**	***Suffix (eg, Jr, III)**	Academic Degrees	Institution	Location (city, state/province, country)	Role or Contribution, eg, chair, principal investigator	Group (if more than 1 Group listed in the byline) and/or Subgroup (eg, Steering Committee)
Roland	Pichler		PhD	Leibniz-Rechenzentrum, Munich, Germany	Munich, Bavaria, Germany	Collaborator	
Heiko	Pfister			Deutsches Herzzentrum München, Institut für Laboratoriumsmedizin, Technische Universität München, Munich, Germany	Munich, Bavaria, Germany	Collaborator	
Paola	Pisano		PhD	Max-Planck-Institut für Biochemie, Munich, Germany	Munich, Bavaria, Germany	Collaborator	
Holger	Prokisch		PhD	Institut für Humangenetik, Technische Universität München, Munich, Germany	Munich, Bavaria, Germany	Co-PI	
Irina	Pugach		PhD	Deutsches Herzzentrum München, Klinik für Herz- und Kreislauferkrankungen, Technische Universität München, Munich, Germany	Munich, Bavaria, Germany	Collaborator	
Lara M	Reimer		PhD	Institut für Informatik, Technische Universität München, Munich, Germany	Munich, Bavaria, Germany	Collaborator	
Michaela	Sander		PhD	Deutsches Herzzentrum München, Institut für Laboratoriumsmedizin, Technische Universität München, Munich, Germany	Munich, Bavaria, Germany	Collaborator	
Veronika	Sanin		MD	Deutsches Herzzentrum München, Klinik für Herz- und Kreislauferkrankungen, Technische Universität München, Munich, Germany	Munich, Bavaria, Germany	PI	
Lea D.	Schlieben		PhD	Institut für Humangenetik, Technische Universität München, Munich, Germany	Munich, Bavaria, Germany	Collaborator	

*First name, last name, and suffix (if applicable) are required and will appear in PubMed.

***First Name and Middle Initial(s)**	***Last Name**	***Suffix (eg, Jr, III)**	Academic Degrees	Institution	Location (city, state/province, country)	Role or Contribution, eg, chair, principal investigator	Group (if more than 1 Group listed in the byline) and/or Subgroup (eg, Steering Committee)
Yannick	Schlote		MSc	Institut Technik – Theologie – Naturwissenschaften, Ludwig-Maximilians-Universität München, Munich, Germany	Munich, Bavaria, Germany	Collaborator	
Sofie	Schmid		MD	Klinikums rechts der Isar, Technische Universität München, Munich, Germany	Munich, Bavaria, Germany	Collaborator	
Raphael S.	Schmieder		MD	Deutsches Herzzentrum München, Klinik für Herz- und Kreislauferkrankungen, Technische Universität München, Munich, Germany	Munich, Bavaria, Germany	Collaborator	
Heribert	Schunkert		MD	Deutsches Herzzentrum München, Klinik für Herz- und Kreislauferkrankungen, Technische Universität München, Munich, Germany	Munich, Bavaria, Germany	Chair, PI	
Marius	Schwab		MD	Deutsches Herzzentrum München, Klinik für Herz- und Kreislauferkrankungen, Technische Universität München, Munich, Germany	Munich, Bavaria, Germany	Collaborator	
Megi	Sharikadze		PhD	Leibniz-Rechenzentrum, Munich, Germany	Munich, Bavaria, Germany	Collaborator	
Ankit	Sinha		PhD	Max-Planck-Institut für Biochemie, Munich, Germany	Munich, Bavaria, Germany	Collaborator	
Fabian	Starnecker		MD	Deutsches Herzzentrum München, Klinik für Herz- und Kreislauferkrankungen, Technische Universität München, Munich, Germany	Munich, Bavaria, Germany	PI	
Medini	Steger		MSc	Max-Planck-Institut für Biochemie, Munich, Germany	Munich, Bavaria, Germany	Collaborator	
Sophia	Steigerwald		MSc	Max-Planck-Institut für Biochemie, Munich, Germany	Munich, Bavaria, Germany	Collaborator	

Supplemental Online Content: Groupauthor Collaborators
*First name, last name, and suffix (if applicable) are required and will appear in PubMed.

***First Name and Middle Initial(s)**	***Last Name**	***Suffix (eg, Jr, III)**	Academic Degrees	Institution	Location (city, state/province, country)	Role or Contribution, eg, chair, principal investigator	Group (if more than 1 Group listed in the byline) and/or Subgroup (eg, Steering Committee)
Ruoyu	Sun		PhD	BioM Biotech Cluster Development GmbH, Martinsried, Germany	Martinsried, Bavaria, German	Collaborator	
Moritz	von Scheidt		MD	Deutsches Herzzentrum München, Klinik für Herz- und Kreislauferkrankungen, Technische Universität München, Munich, Germany	Munich, Bavaria, Germany	Co-Chair, PI	
Matias	Wagner		MSc	Institut für Humangenetik, Technische Universität München, Munich, Germany	Munich, Bavaria, Germany	Collaborator	
Annie	Westerlund		PhD	Deutsches Herzzentrum München, Klinik für Herz- und Kreislauferkrankungen, Technische Universität München, Munich, Germany	Munich, Bavaria, Germany	Collaborator	
Jens	Wiehler		PhD	BioM Biotech Cluster Development GmbH, Martinsried, Germany	Martinsried, Bavaria, German	Co-PI	
Michael	Wierer		PhD	Max-Planck-Institut für Biochemie, Munich, Germany	Munich, Bavaria, Germany	Collaborator	
Peter	Zinterhof		PhD	Leibniz-Rechenzentrum, Munich, Germany	Munich, Bavaria, Germany	Collaborator	
