# Supplementary material for: Ticagrelor vs Prasugrel for Acute Coronary Syndrome in Routine Care
Source: JAMA Netw Open. 2024 Dec 2;7(12):e2448389. doi: 10.1001/jamanetworkopen.2024.48389 (PMC11612834; doi:10.1001/jamanetworkopen.2024.48389)
Supplement: Supplement 3. — Data Sharing Statement [file jamanetwopen-e2448389-s003.pdf]

Data Sharing Statement

Krüger. Ticagrelor vs Prasugrel for Acute Coronary Syndrome in Routine Care. *JAMA Netw Open*. Published December 02, 2024. doi:10.1001/jamanetworkopen.2024.48389

Data
Data available: No

Additional Information
Explanation for why data not available: Health Claims Data are available for qualified researchers on request at OBSERVABLE data base.
